# Impact of Medial Thighplasty on Self-Perception and Body Image After Post-Bariatric Massive Weight Loss

**DOI:** 10.3390/life14111443

**Published:** 2024-11-07

**Authors:** Adrian Matthias Vater, Lennart Erik Schultze-Mosgau, Philipp Edmund Lamby, Matthias Michael Aitzetmüller-Klietz, Karsten Schmidt, Rafael Jakubietz, Michael Jakubietz

**Affiliations:** 1 Klinik für Plastische, Ästhetische and Hand- und Wiederherstellungschirurgie, Klinikum des Universitären, MedizinCampus Niederbayern, Innstraße 76, 94032 Passau, Germany; philipp.lamby@klinikum-passau.de (P.E.L.);; 2 Sektion Plastische und Ästhetische Chirurgie, Klinik und Poliklinik für Unfall-, Hand-, Plastische und Wiederherstellungschirurgie, Universitätsklinikum Würzburg, Oberdürrbacher Straße 6, 97080 Würzburg, Germanyjakubietz_r@ukw.de (R.J.); jakubietz_m@ukw.de (M.J.); 3 Klinik und Poliklinik für Unfall-, Hand-, Plastische und Wiederherstellungschirurgie, Universitätsklinikum Würzburg, Oberdürrbacher Straße 6, 97080 Würzburg, Germany

**Keywords:** body contouring, post-bariatric surgery, skin removal procedures, body dysmorphic disorder, plastic surgery, reconstructive surgery

## Abstract

Background: The demand for body sculpting procedures after massive weight loss (MWL) has grown, with medial thighplasty (MT) emerging as an effective option. This study examines the impact of MT on quality of life (QoL), particularly focusing on body image and self-perception in individuals who have undergone MWL. Methods: This retrospective, single-center study included 21 patients who had post-bariatric MWL and subsequently underwent MT. QoL, with a focus on body image and self-perception, and was assessed through a custom-designed questionnaire administered before and after surgery. Inclusion criteria were a BMI < 35 and a history of bariatric surgery. Results: Twenty-one patients (20 female, 1 male) were included, with an average age of 50.3 years. The median weight loss was 58.4 kg. Post-operatively, the Physical Component Score (PCS-12) showed significant improvement, while the Mental Component Score (MCS-12) did not show a notable change. However, self-acceptance, body contact, sexuality, and self-esteem all significantly improved post-surgery, whereas vitality did not. Conclusions: Consistent with previous studies, MT yields positive outcomes regarding physical well-being. This study further highlights the procedure’s benefits for self-acceptance, body contact, sexuality, and self-esteem. Patients with expectations of improvements in vitality, or mental health concerns like depression or anxiety, should be carefully selected and may benefit from multidisciplinary care, including psychiatry or psychological support, to avoid dissatisfaction with post-surgical outcomes.

## 1. Introduction

Obesity presents a major public health issue in Western countries, significantly increasing the risk of comorbidities, such as cardiovascular disease, type 2 diabetes, and musculoskeletal disorders [1,2]. While lifestyle modifications and dietary changes are first-line approaches, they often fail to produce long-lasting weight reduction in individuals with morbid obesity. Bariatric procedures, such as Roux-en-Y gastric bypass or sleeve gastrectomy, have proven to be effective solutions, enabling substantial and sustained weight loss [3]. As the demand for bariatric surgery increases, the benefits of MWL, including improved health and increased life expectancy, become more apparent [4].

However, MWL often leads to the unintended consequence of excess skin, particularly in areas like the abdomen, arms, and thighs. This skin laxity can result in functional impairments, hygiene difficulties, and an increased risk of skin infections [5]. Additionally, excess skin negatively impacts body image, leading to psychological distress, decreased self-esteem, and dissatisfaction with one’s post-weight loss appearance. These challenges have driven an increased demand for body contouring procedures to address both the physical and aesthetic issues that arise following MWL [6,7,8,9,10].

Body contouring surgeries, including abdominoplasty, brachioplasty, and circumferential body lifts, have been extensively studied in terms of their physical and psychological outcomes. However, these studies often categorize body contouring procedures as a whole, overlooking the specific effects of individual surgeries on QoL and body image [7]. One procedure that has received less attention is MT, which addresses excess skin and soft tissue in the inner thighs. This procedure is especially relevant for patients who have experienced MWL, as the thighs are a common area of concern, yet underrepresented in the literature.

MT involves the removal of excess skin and fat from the inner thighs to reshape and improve the contour, providing both aesthetic and functional benefits. Despite its complexity and the potential risks involved, MT has demonstrated promising results, enhancing body image and self-esteem in patients post-MWL. However, limited data exist regarding its psychological benefits, especially in terms of body image and mental health outcomes [8].

Excess skin in the inner thighs can lead to functional problems, such as skin irritation, difficulty maintaining hygiene, and discomfort during physical activities or when wearing certain clothing. MT addresses these issues by surgically removing the excess skin, thereby improving thigh contours and enhancing mobility and comfort [9]. Studies have shown that MT can reduce skin irritation, make daily activities easier, and improve physical exercise comfort, ultimately leading to a better QoL [10,11,12]. By alleviating physical discomfort, MT also encourages patients to re-engage in social and physical activities without embarrassment or limitations [13].

In addition to the physical benefits, body image dissatisfaction remains prevalent in individuals who have undergone MWL due to concerns about excess skin [14]. This dissatisfaction can lead to poor self-esteem, heightened self-consciousness, and reduced sexual confidence, which in turn may contribute to anxiety and depression. For many patients, the goal of MT extends beyond improving physical appearance; they seek to boost self-confidence and psychological well-being [15].

Preliminary evidence suggests that MT can significantly enhance body image perception, reducing feelings of shame or anxiety related to physical deformities and improving self-esteem. Many patients report increased satisfaction with their post-operative body image, which positively influences their social and intimate relationships [16].

This study aims to fill the gap in the literature by evaluating the psychological impact of MT on individuals who have experienced MWL following bariatric surgery.

Specifically, we assess changes in areas such as vitality, self-acceptance, body contact, sexuality, and self-esteem using validated QoL questionnaires. By focusing on MT alone, rather than on body contouring as a broad category, this study offers a more nuanced understanding of the procedure’s physical, psychological, and social outcomes. We hypothesize that MT not only enhances physical comfort and mobility but also significantly improves psychological well-being, particularly in relation to body image and patient satisfaction.

In summary, this study aims to provide critical insights into the effects of MT on body image and mental health outcomes following MWL. By focusing on this specific procedure, we hope to highlight the psychological and social benefits that extend beyond the physical improvements typically associated with body contouring. Through a detailed analysis of post-operative outcomes, we aim to contribute to the broader understanding of how targeted surgical interventions can significantly enhance QoL for patients dealing with excess skin after significant weight loss.

## 2. Material and Methods

In this retrospective study 21 patients who underwent MT following MWL after bariatric surgery between 2009 and 2015 were analyzed. All procedures were performed by the same surgical team at the University Hospital of Würzburg to maintain consistency in surgical technique and post-operative care. The study aimed to evaluate both physical and psychological outcomes in patients dealing with excess skin and body image dissatisfaction after MWL.

### 2.1. Patient Selection and Data Collection

Patients were included if they experienced MWL following bariatric surgery and underwent MT using the vertical scar technique. Bariatric procedures included Roux-en-Y gastric bypass and sleeve gastrectomy. Patients were required to have a BMI < 35 for at least six months pre-operatively. Data, including age, sex, total weight loss, prior surgeries, and comorbidities, were collected from medical records. Patients with pre-existing mental illness, incomplete surgical data, or inadequate questionnaire responses were excluded to ensure result validity.

### 2.2. Surgical Technique

All patients underwent MT using the vertical scar technique, known for effectively contouring the medial thigh by removing redundant skin and fat while minimizing visible scarring [17]. This technique was chosen for its ability to address both functional and aesthetic concerns, particularly in patients with significant skin laxity after MWL.

### 2.3. Quality of Life (QoL) Assessment

#### 2.3.1. QoL Was Evaluated Using Three Validated Tools

*Short Form Health Survey-12 (SF-12):* Measures physical and mental health across eight domains, generating the Physical Component Summary (PCS) and Mental Component Summary (MCS) scores [18].

*Dresden Body Image Score-35 (DKB-35):* A 35-item questionnaire assessing body image and self-perception in areas like vitality, self-acceptance, body contact, sexuality, and self-esteem [19].

*Patient Health Questionnaire-4 (PHQ-4):* A brief tool for screening symptoms of depression and anxiety [20].

#### 2.3.2. Data Analysis

Statistical analyses were conducted using IBM SPSS Statistics Version 29.0.2.0. Continuous variables were reported as means, and categorical data as frequencies and percentages. Paired *t*-tests were used to compare pre-operative and post-operative QoL scores. A *p*-value of less than 0.05 was considered statistically significant.

### 2.4. Ethical Considerations

This study adhered to ethical standards in line with the Declaration of Helsinki. Informed consent was obtained from all participants, and the study was approved by the local ethics committee.

## 3. Results

A total of 21 patients (20 females and 1 male) were included in the analysis. The mean age of participants was 50.3 years, with females having a mean age of 49.3 years and the single male participant being 70 years old. The median weight loss across all patients was 58.4 kg, with females losing an average of 53.3 kg and the male patient achieving a weight loss of 85 kg. The median follow-up period was 9.77 years, ensuring that both short- and long-term outcomes were captured. Notably, four patients got married during the period between their surgery and the completion of the study’s questionnaires, highlighting potential social improvements post-operatively.

Complications reported included delayed wound healing in 11 cases, infection requiring surgical revision in one case, post-operative seroma in 7 cases, and excessive scarring observed in one case.

### 3.1. Physical Outcomes (SF-12)

**Figure 1:**
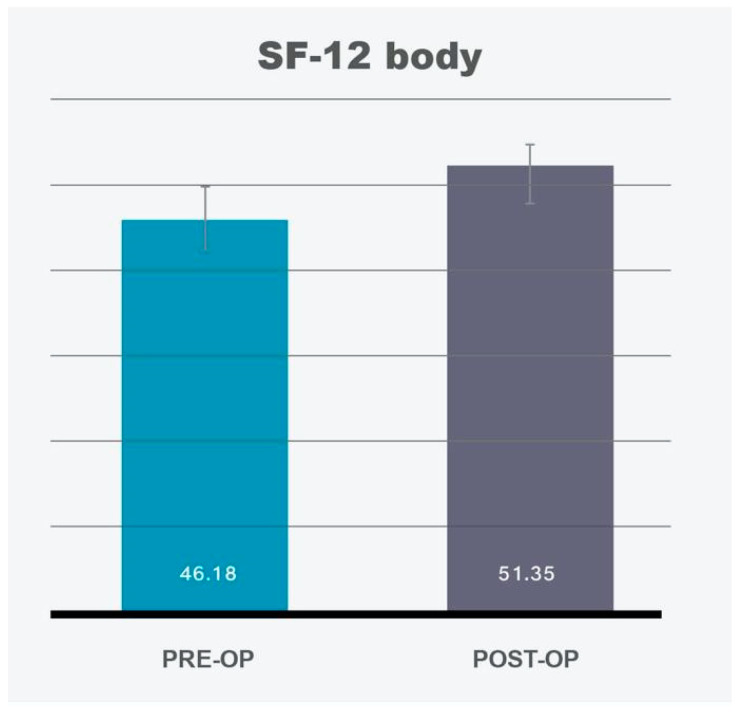
SF-12 Questionnaire: SF-12 body shows significant improvement (*p* = 0.011). 95% confidence intervals are depicted by vertical bars.

**Figure 2:**
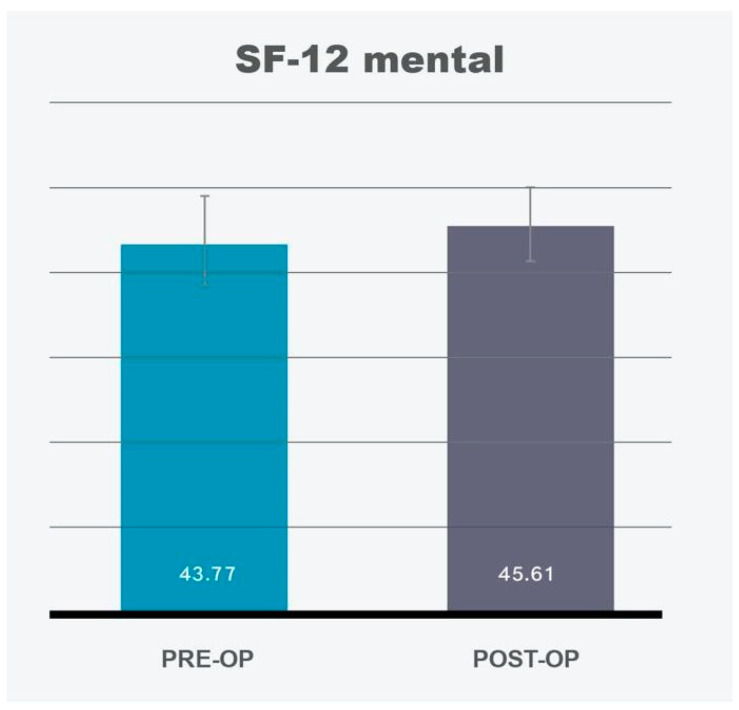
SF-12 Questionnaire: SF-12 mental scale does not show any significant change (*p* = 0.277). Confidence intervals of 95% are depicted by vertical bars.

The analysis of SF-12 data revealed a significant improvement in the Physical Component Summary (PCS-12) post-operatively (Figure 1, Table 1), consistent with prior studies demonstrating improvements in physical functioning following medial thighplasty (MT). Patients reported increased mobility, decreased physical discomfort, and enhanced ability to perform daily activities without limitations caused by excess skin. However, the Mental Component Summary (MCS-12) did not show any significant change between the pre-operative and post-operative assessments (Figure 2, Table 1).

Standard Deviation (SD) for PCS-12 was 9.57; SD for MCS-12 was 14.02.

These results suggest that, while MT leads to improved physical quality of life (QoL), the procedure alone may not be sufficient to drive significant improvements in broader mental health outcomes.

### 3.2. Body Image Outcomes (DKB-35)

**Figure 3:**
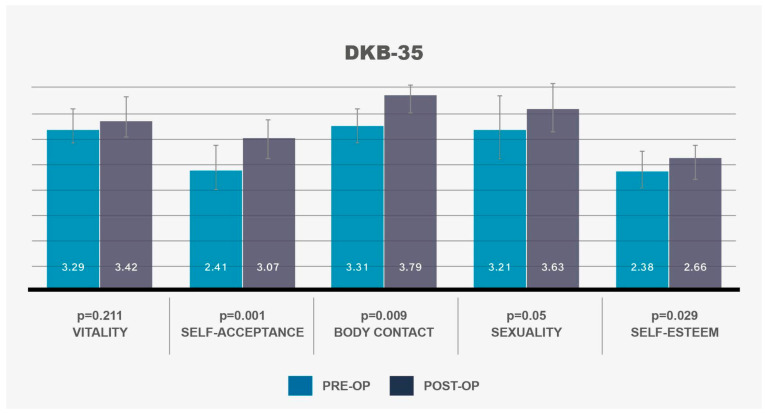
Body image scale (DKB-35). Significant improvement is displayed in self-acceptance, body contact, sexuality, and self-esteem. No significant change in vitality. Confidence intervals of 95% are depicted by vertical bars.

The DKB-35 data demonstrated significant improvements across most dimensions of body image perception. Post-operatively, patients reported better self-acceptance (SD 0.89), enhanced comfort with body contact (SD 0.85), improved sexual confidence (SD 1.12), and higher self-esteem (SD 0.65) (Figure 3, Table 1). These results underline the positive psychological effects of MT concerning body image and self-perception. However, the vitality dimension did not show a statistically significant increase post-operatively, indicating that, while patients may feel better about their appearance, this does not necessarily translate to increased energy or liveliness in daily life.

### 3.3. Mental Health Outcomes (PHQ-4)

**Figure 4:**
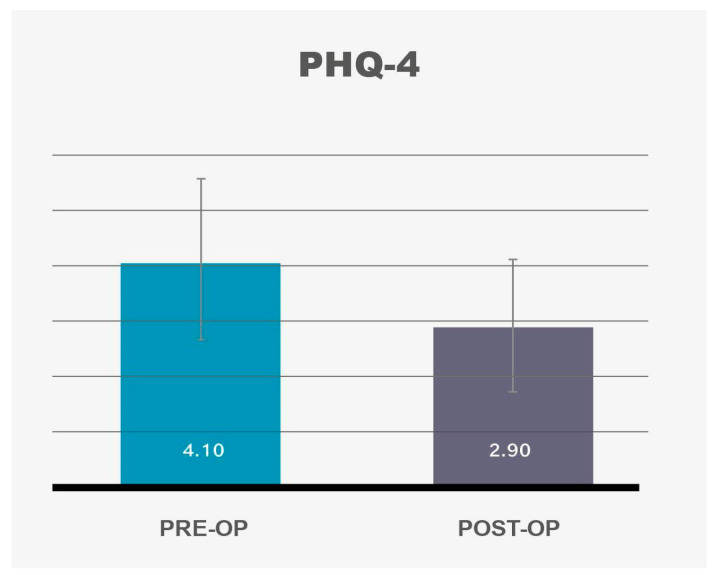
PHQ-4 depression scale shows no significant change post-operatively (*p* = 0.068). Confidence intervals of 95% are depicted by vertical bars.

The analysis of PHQ-4 scores revealed no significant changes in symptoms of depression or anxiety post-operatively (SD 3.52) (Figure 4, Table 1). Despite the positive body image outcomes reported in the DKB-35, the lack of significant improvement in PHQ-4 scores suggests that MT may not have a direct impact on underlying mental health conditions, such as depression or anxiety. These findings align with the results of the MCS-12, further emphasizing that, while MT can address body image concerns, it may not be a comprehensive solution for all aspects of psychological well-being.

**Table 1:** Baseline data and results from SF-12, DKB-35, and PHQ-4 questionnaires.

Patient Characteristics	PRE-OP		POST-OP
*n*		21	
male		1	
female		20	
mean age (years)		50.3	
		(f 49.3 | m 70)	
mean follow-up time (years)		9.77	
mean weight loss (kg)		58.4	
		(f 53.3 | m 85)	
SF-12 body	46.18		51.35
SF-12 mental	43.77		45.61
DKB-35 vitality	3.29		3.42
DKB-35 self-acceptance	2.41		3.07
DKB-35 body contact	3.31		3.79
DKB-35 sexuality	3.21		3.63
DKB-35 self-esteem	2.38		2.66
PHQ-4	4.10		2.90

## 4. Discussion

Obesity is a prevalent issue in Western societies, often prompting individuals to seek bariatric surgery to address health concerns [1]. Following successful bariatric surgery and subsequent massive weight loss (MWL), many patients request body contouring procedures to address excess skin and achieve their desired body shape [21]. These transformations significantly impact QoL and body image [9,10,11,12,13,14,15,16]. Perceptions, thoughts, and feelings about one’s body play a crucial role in overall well-being and QoL. Understanding how body sculpting procedures influence body image and self-perception is essential for optimizing post-bariatric surgery outcomes and providing comprehensive patient care.

MT represents a valuable intervention for individuals who have undergone MWL, offering significant improvements in the physical aspects of QoL [9,10,11,12,13]. When consulting with a plastic surgeon, it becomes evident that this patient collective has high expectations regarding the improvement of their overall body image [22]. To address these concerns early, it is important to discuss how MT can lead to significant improvements in thigh contour and aesthetics in general. While MT can enhance the appearance of the thighs, it does not necessarily improve body contact sensation in every case and can even lead to complications [23,24]. It is crucial to recognize that it is just one component of a comprehensive body sculpting strategy. Unrealistic expectations regarding the extent of improvement in overall body image through a single procedure can lead to dissatisfaction and psychological distress post-operatively.

Several factors contribute to patients’ unrealistic expectations. Media portrayals of cosmetic surgery procedures often emphasize dramatic transformations and flawless outcomes, creating unrealistic standards for appearance [25]. Additionally, patients may have internalized societal norms and ideals of beauty, expecting perfection from surgical interventions. Furthermore, the emotional and psychological toll of living with excess skin after MWL can amplify patients’ desire for a “perfect” body, leading them to overestimate the transformative power of surgical procedures [26].

While previous studies have shown lower satisfaction rates after MT compared to other body contouring procedures [24], the data in this study indicate a positive impact on physical QoL and body perception. Clear differences in changes in physical and mental QoL can be seen in the SF-12 questionnaire results. However, significant improvements in QoL following body contouring surgeries have consistently been demonstrated [27,28]. In this study, alongside the positive impact of MT on physical QoL, the lack of improvement in mental QoL was also confirmed. The primary goal of body contouring surgeries is to eliminate the physical limitations associated with excess skin folds.

Coriddi et al. documented significant improvements in various functional aspects, such as difficulties with personal hygiene, finding suitable clothing, skin irritation, and pain in the neck and abdominal areas in a study involving 49 patients who underwent abdominoplasty after MWL [29]. Singh et al. compared the mental and physical QoL of post-bariatric patients who underwent body contouring surgeries with those who did not wish to undergo such procedures. They found that patients who opted out of body contouring had a significantly higher mental QoL. This was attributed to their already high mental QoL, reducing their motivation for further surgery. The authors suggested that underlying psychological factors in the body contouring cohort could not be altered by surgery. This sampling bias and unmet patient expectations are also likely to have been present in this study [30].

In this study, MT significantly impacted body image, particularly influencing self-acceptance, body contact, sexuality, and self-esteem. As individuals see their body contours become smoother and more proportionate, they often experience a boost in self-perception. This positive shift leads to a significant increase in self-acceptance. The removal of uncomfortable excess skin can alleviate physical discomforts, such as chafing and infections, making individuals feel more at ease and open to physical contact in various forms, including social, platonic, and intimate interactions.

Moreover, body image plays a crucial role in one’s sexuality. An enhanced appearance of the thighs can make individuals feel more attractive and confident in their sexuality. This newfound sexual confidence can lead to a more satisfying sex life, as individuals feel less inhibited and more engaged during intimate encounters. Achieving a desired physical appearance can make individuals feel more in control of their appearance. This newfound sense of control often translates to increased assertiveness and positivity, permeating various aspects of life, including personal relationships and professional interactions.

In a prospective, multicenter study, Mocquard et al. examined the sexual QoL of 49 post-bariatric, post-MT patients using the Female Sexual Function Index (FSFI) [13]. They found no improvement in sexual quality of life. The FSFI is specifically designed to assess various aspects of sexual function in women, including sexual desire, arousal, lubrication, orgasm, satisfaction, and pain during sexual activity, measuring the functionality and quality of sexual experiences.

In contrast, the sexuality-related questions in DKB-35 focus on body image perception and how this perception influences self-esteem and emotional well-being. The DKB-35 primarily assesses satisfaction with one’s body and the evaluation of one’s physical appearance. While this can have indirect effects on sexuality, such as sexual confidence or the sense of sexual attractiveness, the DKB-35 does not directly measure sexual functions or dysfunctions. Sexuality encompasses not only the act itself but also emotional intimacy, physical intimacy, and communication. Therefore, it is important to question whether the FSFI is the appropriate tool for assessing overall sexual QoL.

Reasons for these results are manifold. MWL can lead to significant changes in the body beyond just excess skin. It can affect muscle tone, tissue elasticity, and overall body composition. MT primarily addresses aesthetic concerns rather than functional improvements in the thighs. It does not enhance muscle function, cardiovascular health, or metabolic rate. Since the procedure does not improve muscle strength, endurance, or cartilage thickness, it has little effect on the physical components of vitality. Additionally, vitality is closely linked to long-term lifestyle choices, such as diet, exercise, and sleep, which MT does not directly impact. For lasting improvements in vitality, individuals need to adopt comprehensive lifestyle changes, including regular physical activity and proper nutrition, beyond the scope of cosmetic surgery.

When evaluating the impact of a vertical incision technique in medial thighplasty on post-operative outcomes, it is important to consider both functional and aesthetic results, as well as the potential psychological benefits. According to a study by Losco et al., the “Helix Thigh Lift” technique, which involves a vertical incision, may provide improved contouring for patients with severe deformities following MWL [31]. This technique allows for more effective removal of redundant skin and fat, particularly in the longitudinal axis of the thigh, leading to enhanced overall thigh shape and a more aesthetically pleasing outcome. The study suggests that these contouring improvements could potentially lead to higher patient satisfaction with their physical appearance post-operatively.

**Figure 5:**
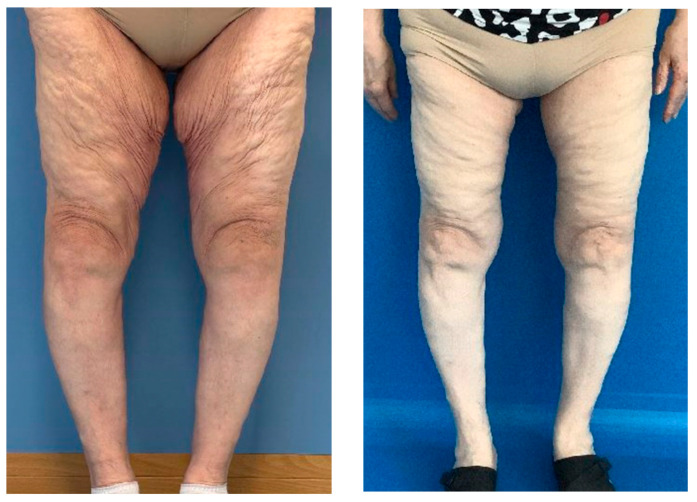

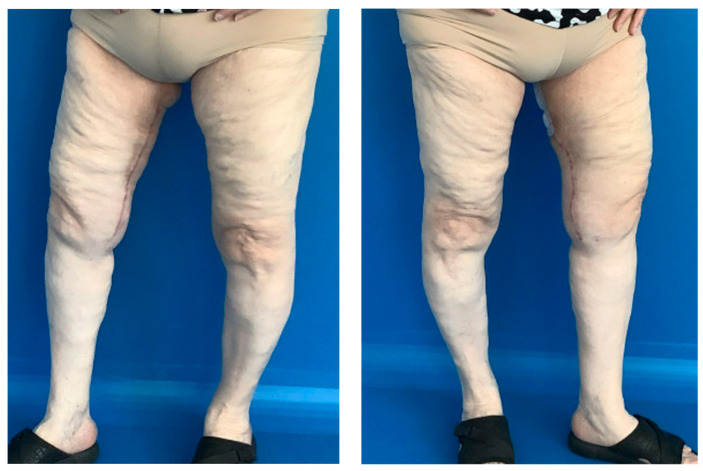
Results pre-operative and six weeks post-operative in frontal and oblique view.

However, the question remains as to whether these contouring improvements necessarily lead to a better body image and QoL. The study by Gusenoff et al. on MT in the MWL population evaluates the potential outcomes and complications of such procedures [32]. While vertical incision techniques may offer superior contouring results (Figure 5), complications can arise, including prolonged recovery times and increased scarring. The long-term implications of these outcomes on body image and QoL should be carefully weighed.

In summary, this study demonstrates that medial thighplasty following MWL has a significant impact on physical QoL and certain aspects of body image perception. While physical improvements are evident, mental health outcomes did not show significant enhancement. This reinforces the need for comprehensive pre-operative counseling and psychological support for patients undergoing body contouring procedures. The expectation that surgical interventions can completely transform one’s mental health may lead to disappointment and dissatisfaction. Therefore, it is essential to adopt a holistic approach that addresses both physical and psychological factors, ensuring that patients have realistic expectations and access to the necessary support throughout their journey [33,34,35,36,37].

This study has several limitations. Firstly, the retrospective design may introduce selection bias, as only patients who underwent MT were included. Secondly, the relatively small sample size limits the generalizability of the findings. Additionally, the follow-up period may not have been long enough to capture the full psychological benefits of the procedure.

Future studies should consider larger, multicenter designs and longer follow-up periods to better assess the long-term outcomes of MT. Incorporating qualitative assessments may provide deeper insights into patient experiences and satisfaction post-surgery.

## 5. Conclusions and Learning Points

In conclusion, MT after MWL demonstrates significant improvements in physical health and various aspects of body image and self-esteem. These findings highlight the importance of addressing both physical and psychological concerns in patients seeking body contouring procedures. A holistic approach to post-MWL care, incorporating psychological support, is essential to optimize patient outcomes and satisfaction.

## Data Availability

All patient data were anonymized and received by the patient chart with the consent of the local ethics committee.
